# Pregnancy-related atypical hemolytic uremic syndrome with renal, cardiac and obstetric complications and a satisfactory recovery: a case report

**DOI:** 10.1080/0886022X.2021.1893187

**Published:** 2021-03-03

**Authors:** Emrah Gunay, Mahsum Ozan, Seyhmus Kaya, Ece Ocal, Zeynep Kutlu, Ayhan Senol, Ramazan Danis, Erkan Baysal, Burhan Sami Kalin, Huseyin Derya Dincyurek, Cengiz Demir

**Affiliations:** a Department of Nephrology, Health Sciences University of Turkey, Diyarbakir Gazi Yasargil Training and Research Hospital, Diyarbakir, Turkey; b Department of Internal Medicine, Health Sciences University of Turkey, Diyarbakir Gazi Yasargil Training and Research Hospital, Diyarbakir, Turkey; c Department of Pathology, Health Sciences University of Turkey, Diyarbakir Gazi Yasargil Training and Research Hospital, Diyarbakir, Turkey; d Department of Perinatology, Health Sciences University of Turkey, Diyarbakir Gazi Yasargil Training and Research Hospital, Diyarbakir, Turkey; e Department of Interventional Radiology, Health Sciences University of Turkey, Diyarbakir Gazi Yasargil Training and Research Hospital, Diyarbakir, Turkey; f Department of Cardiology, Health Sciences University of Turkey, Diyarbakir Gazi Yasargil Training and Research Hospital, Diyarbakir, Turkey; g Department of Intensive Care, Health Sciences University of Turkey, Diyarbakir Gazi Yasargil Training and Research Hospital, Diyarbakir, Turkey; h Department of Haematology, Health Sciences University of Turkey, Diyarbakir Gazi Yasargil Training and Research Hospital, Diyarbakir, Turkey

Dear Editor,

A typical hemolytic uremic syndrome (aHUS), which occurs with the uncontrolled activity of the complement system, can occur at any age. Triggers of aHUS include pregnancy, drug use, autoimmune diseases, transplantation, cobalamin deficiency, and malignancy [1]. Pregnancy-related aHUS (P-aHUS) mostly occurs during the postpartum period, but it can be seen in all trimesters [2]. Extrarenal manifestations are rare. aHUS-related cardiomyopathy, characterized by sudden systolic heart failure, can occur in 10% of the aHUS patients [3]. We present a happy-ending story of a 19-year-old female patient with P-aHUS complicated by severe renal failure, a preterm birth, and postpartum cardiomyopathy.

A 19-year-old woman without any chronic disease was admitted to the intensive care unit at the 23rd week of pregnancy due to fever and hypotension. Serum creatinine and platelet values measurements from 18 days before her admission were 0.61 mg/dL and 284 × 10^3^/μL, respectively. At her admission, these values were 1.76 mg/dL, and 65 × 10^3^/μL, respectively (Table 1). Schistocyte were seen in the peripheral smear. After a blood sample was taken for ADAMTS-13, plasma exchange (PEX) treatment was started due to a diagnosis of thrombotic microangiopathy (TMA). Thrombocytopenia and kidney injury did not improve with seven sessions of PEX. Antinuclear antibodies, anti-double-stranded DNA, antineutrophil cytoplasmic antibodies (p-ANCA and c-ANCA), C3, and C4 values of the patient were all within normal limits. Direct coombs were negative. Haptoglobulin could not be checked. Hemodialysis was started due to the progression of renal failure and the development of hypervolemia. A kidney biopsy was performed, and TMA findings were reported. Since she was not hypertensive during her pregnancy and her liver enzymes did not exceed the upper limit of normal, preeclampsia (PE)/Eclampsia and HELLP syndrome were not considered as diagnoses. Thrombotic thrombocytopenic purpura (TTP) was ruled out due to the patient’s normal ADAMTS-13 level (41.8%). Therefore, P-aHUS was accepted as the diagnosis. Due to the detection of oligohydramnios and fetal distress in a Doppler ultrasonography and a non-stress test, the patient was taken for an emergent cesarean at 25 weeks of gestation, and the planned start of the eculizumab treatment was delayed for 72 h in case of spontaneous recovery after birth. The patient was taken to intensive care with a sudden onset of respiratory failure at the 60th hour post-partum. On echocardiogram (ECHO), her left ventricular ejection fraction (LVEF) was 40–45%, and global hypokinesia of all heart walls was reported. Massive pulmonary effusion was seen on a thorax ultrasonography. Hemodialysis and ultrafiltration (UF) were started, and after 7.5 L of UF in eight hours, respiratory failure improved. Eculizumab treatment was started, and the patient’s clinical condition improved during follow-up. DNA sequence analysis detected no disease-related variant in the analyzed regions of the complement factor H, I and complement factor H-related-5 genes. Eight doses of eculizumab were given in the first three months after birth, after which eculizumab treatment could not be continued because the patient did not return to the hospital for personal reasons. At the seventh month postpartum, the patient was not given eculizumab for the last four months. Her physical examination was completely normal, serum creatinine, estimated glomerular filtration rate (e-GFR) and thrombocyte values were 1.06 mg/dL, 76 mL/min and 230 × 10^3^/μL, respectively. At that time, LVEF in ECHO was 55%, and wall movements were normal. Since her genetic tests were negative and satisfactory recovery is achieved, close follow-up without eculizumab is planned for the patient.

**Table 1.** Clinical and laboratory results.

Date/event	Cre	GFR	Hb	Plt	ALT	Alb	ECHO
Hospitalisation Day: 1	1.76	41	10.5	95	38	2.7	
Day: 4 Plazma exchange (PEX) started	2.2	31	10.7	72	29	2.3	
Day: 12 Hemodialysis(HD) – 2 hours PEX – 6th	3.75	16	9.2	139	9	2.2	LVEF: %60, all wall movements are normal
Day: 13 HD – 3 hours PEX – 7th (The last)	3.0	21	8.8	120	17	2.7	
Day: 14 HD – 3 hours ES replacement (2 units)	2.42	28	8	121	15	2.7	
Day: 16 Kidney biopsy	2.36	29	10	110	19	2.5	
Day: 19 Birth with CS (25th week of pregnancy; oligohydramnios and fetal distress occured)	2.98	22	9.6	95	12	2.6	
Day: 22 Acute pulmonary edema Transfer to ICU Urgent HD + 7.5 L UF Eculizumab 900 mg/week(1st dose) ES replacement (2 units)	2.71	24	10.7	120	10	2.9	LVEF: %40–45, global hypokinesia of all heart walls
Day: 29 Eculizumab 900 mg/week (2nd dose)	3.05	21	9.8	137	17	3.1	LVEF: %35–40. global hypokinesia of all heart walls
Day: 36 Eculizumab 900 mg/week (3rd dose) Discharge	2.54	26	9.8	236	13	3.3	
Day: 51 Eculizumab 1200 mg/2 weeks (5th dose)	2.91	22	10.4	239	15	3.4	LVEF: %45–50. global mild hypokinesia of all heart walls
Day: 79 Eculizumab 1200 mg/2 weeks (7th dose)	1.76	41	10.7	317	8	3.2	
Day:97 Eculizumab 1200 mg/2 weeks (8th dose, the last dose)	1.43	53	12.1	245	11	3.5	LVEF: %50. global mild hypokinesia of all heart walls
Day: 183	1.1	72	12.3	191	9	NA	LVEF: %55. all wall movements are normal
Day: 235	1.06	76	14.3	230	11	NA	

Cre: Serum creatinine (mg/dL); GFR: estimated glomerular filtration rate (ml/min); Hb: Hemoglobine (g/dL); Plt: Platelet (×10^3^/μL); Ast: Aspartate aminotransferase (U/L); Alt: Alanine aminotransferase (U/L); Alb: Serum albümine (g/dL); ECHO: Echocardiogram; LVEF: Left ventricular ejection fraction ES: Erythrocyte suspension; ICU: Intensive care unit; UF: Ultrafiltration; CS: Cesarean section; NA: Non-applicable.

Differential diagnoses and treatments of TMAs during pregnancy are difficult to manage and require a multidisciplinary approach. P-aHUS, TTP, PE/Eclampsia and HELLP syndrome may mimic each other. Moreover, these diseases can be complicated by each other [2]. The benefit of plasma treatments has only been shown in immune ADAMTS-13 deficiency-related TTP. When there is no haematological and renal improvement in the first four or five PEX treatments, the treatment should be based on the patient’s ADAMTS-13 level. In cases of significant deficiency, the diagnosis of TTP is confirmed, and it may be necessary to continue PEX and intensify immunosuppressive therapy. Normal ADAMTS-13 levels support the diagnosis of aHUS. In this case, PEX should be discontinued and Eculizumab should be started [4]. Early initiation of eculizumab may minimize renal damage [2,5]. Some authors accept the threshold values for ADAMTS-13 as 10% or 20% [2,4].

Lack of a pathognomonic test to diagnose aHUS along with reluctance to give such an expensive treatment may cause some hesitations regarding the initiation of the treatment. These delays can cause permanent renal damage or serious extra-renal complications, such as cardiomyopathy. Merril et al. stated in their study that they preferred early initiation of eculizumab and, after achieving remission, close follow-up and discontinuation of the drug. The low relapse rates may be related to the low complement factor H mutations in the patient cohorts [6]. Fakhouri et al. reported in their prospective study that discontinuation of eculizumab is reasonable and safe, in accordance with the complement mutation results of the patients [7]. The presence of complement gene abnormalities is a risk for aHUS relapse after anti-C5 treatment discontinuation, including in P-aHUS patients. In an international study group report, it is recommended to discontinue eculizumab treatment after the usage minimum of six months in P-aHUS patients [2].

Acute systolic heart failure is one of the most serious complications in patients with aHUS. After 7.5 L of UF in eight hours, our patient’s pulmonary edema improved. It is not correct to make a final decision as to how many liters to apply when starting UF in these patients. Within the limits allowed by the patient’s haemodynamics, UF should be continued until clinical relief is achieved. Cardiomyopathy may improve with eculizumab and other treatments.

In conclusion, it should always be kept in mind that the pregnancy process is a triggering factor for aHUS. Eculizumab should be started immediately if the ADAMTS-13 level is greater than 10% in patients who do not recover after four or five sessions of PEX. Discontinuation of eculizumab with a strict follow-up protocol may be considered in aHUS cases where genetic tests are negative, the triggering factor is eliminated, and after clinical and laboratory improvement has occurred.
